# Reconstructive Strategies in Post-Traumatic Osteomyelitis of the Lower Limb: A Case Series and Surgical Algorithm Analysis

**DOI:** 10.3390/jcm14196746

**Published:** 2025-09-24

**Authors:** Marta Jagosz, Piotr Węgrzyn, Michał Chęciński, Maja Smorąg, Jędrzej Króliński, Szymon Manasterski, Patryk Ostrowski, Ahmed Elsaftawy

**Affiliations:** 1 Department of Plastic and Hand Surgery, St. Jadwiga Śląska Hospital, 55-100 Trzebnica, Poland; marta.malgorzata.jagosz@gmail.com (M.J.); wengrzu@gmail.com (P.W.); michal.checinski@gmail.com (M.C.); maja.smorag@gmail.com (M.S.); jedrzejkrolinski@gmail.com (J.K.); smanasterski@gmail.com (S.M.); 2 Department of Anatomy, Jagiellonian University Medical College, 31-008 Kraków, Poland; ostrowskipatryk0@gmail.com; 3 Youthoria, Youth Research Organization, 31-008, Kraków, Poland

**Keywords:** fasciocutaneous flap, free flap transfer, infection, limb salvage, microsurgery, muscle flap, orthopedic, post-traumatic osteomyelitis, tibial infection

## Abstract

**Background:** Post-traumatic osteomyelitis (PTO) of the lower extremity is among the most demanding problems in orthoplastic reconstructive surgery. It typically follows open fractures, failed osteosynthesis, or implant infection. Effective management requires coordinated infection control, stable skeletal fixation, and timely vascularized soft-tissue coverage. **Methods:** We conducted a retrospective case series of 20 consecutive patients with PTO of the lower limb treated between 2021 and 2024 at a tertiary orthoplastic center. All patients underwent radical debridement, culture-directed intravenous antibiotic administration, and soft-tissue reconstruction using local muscle, fasciocutaneous, or free flaps; vascularized bone flaps were used to select nonunion cases. The primary outcomes were flap survival, complications, infection resolution, and limb salvage. Exploratory analyses included descriptive subgroup summaries by flap category. **Results:** Among 20 patients (15 men, 5 women; mean age 53.6 years), reconstructions included reverse/pedicled sural flaps (n = 9), hemisoleus muscle flaps (n = 7), medial gastrocnemius muscle flaps (n = 2), peroneus brevis muscle flaps (n = 2), and free flaps (n = 6), which comprised anterolateral thigh (ALT), medial femoral condyle (MFC) osteoperiosteal, deep circumflex iliac artery (DCIA) osteocutaneous, and radial forearm free flaps (RFFFs). Single-flap reconstructions were performed in 13 cases, whereas multistage/multiflap strategies were used in 7. Overall flap survival was 90%. Major flap complications comprised partial necrosis in two reverse sural flaps and one complete loss of a reverse sural flap; two patients had minor wound dehiscence. Infection resolved in 18/20 patients (90%; 95% CI ≈ 0.70–0.97). One patient requested below-knee amputation due to persistent nonunion associated with a pathological fracture. At a mean 10-month follow-up, all limb-salvaged patients were ambulatory. **Conclusions:** Effective reconstruction of PTO is improved by using a patient-specific algorithm that considers the defect location, vascular status, and host comorbidities. Local muscle and fasciocutaneous flaps remain dependable for most defects, with free or vascularized bone flaps reserved for composite or recalcitrant cases. Early referral to high-volume centers, radical debridement, and orthoplastic collaboration are critical for optimizing limb salvage. Our findings should be interpreted in light of the study’s retrospective design and small sample size.

## 1. Introduction

Post-traumatic osteomyelitis (PTO) remains a severe, function-limiting complication of high-energy lower-limb injuries, particularly open tibial fractures. Incidence ranges from ~1% in closed fractures to >30% in Gustilo–Anderson type IIIB/C injuries and is magnified by delayed coverage, repeated debridements, or compromised host factors [1,2]. The pathogenesis is multifactorial and can include devitalized bone and soft tissues, impaired perfusion, biofilm formation on hardware, and inadequate source control, all of which contribute to sustaining chronic infection. In infections, *Staphylococcus aureus* (including methicillin-resistant strains MRSA) predominates, with polymicrobial flora common in long-standing cases [3,4].

Managing PTO of the tibia is particularly challenging due to the subcutaneous location of the bone and limited soft tissue envelope. The decision-making process must address both infection eradication and anatomical reconstruction. While antibiotics are essential, their efficacy is limited without surgical debridement and viable tissue coverage. The role of vascularized soft tissue in delivering immune cells and systemic antibiotics to the infected area has been extensively documented [4].

The principles of care include radical debridement, culture-directed antibiotics, skeletal stability, and vascularized soft-tissue coverage. Early soft-tissue reconstruction reduces infection and improves union and function compared to delayed coverage, as established by Godina [5] and reaffirmed in contemporary series and meta-analyses (while acknowledging the evolving windows in modern practice).

This study adds to the existing algorithms, which often emphasize defect location alone. Our model explicitly integrates (i) defect location, (ii) limb vascular status (CTA/duplex), and (iii) host factors (diabetes, peripheral arterial disease, smoking) into flap selection and sequencing. We also detail composite reconstructions combining multiple regional flaps where free-flap resources are constrained. Additionally, we codify indications for vascularized osteocutaneous flaps (MFC, DCIA) within PTO care approaches that are described in focused reports but under-represented in PTO algorithms.

Our objective is to report institutional outcomes using this algorithm, describe complication patterns by flap category, and contextualize out results in the context of the literature on PTO reconstruction, timing, and adjuncts. We additionally outline limitations and directions for future comparative work.

## 2. Materials and Methods

### 2.1. Study Design and Setting

This was a retrospective case series from a high-volume tertiary orthoplastic center. Consecutive adult patients treated for lower-limb PTO from January 2021 through December 2024 were identified from a prospectively maintained surgical database (Table 1).

The table summarizes demographic data, injury etiology, anatomical site of the defects, reconstructive flap techniques applied (anterolateral thigh flap “ALT”, medial femoral condyle flap “MFC”, deep circumflex iliac artery bone flap “DCIA”, radial forearm free flap “RFFF”), and any associated complications in the cohort of 20 patients.

### 2.2. Inclusion and Exclusion Criteria

This study focused exclusively on patients with (i) radiological and microbiological confirmation of PTO; (ii) open soft-tissue defects with exposed bone and/or hardware; and (iii) a minimum of 6 months postoperative follow-up.

Patients with hematogenous osteomyelitis, prohibitive comorbidity precluding surgery (e.g., NYHA IV heart failure), incomplete records, or loss to follow-up were excluded.

### 2.3. Preoperative Evaluation

All patients underwent a comprehensive preoperative assessment designed to map the extent of infection, evaluate surgical risks, and guide reconstructive planning. Laboratory investigations included standard inflammatory markers—(i) erythrocyte sedimentation rate (ESR), (ii) C-reactive protein (CRP), and (iii) white blood cell count (WBC)—which together provided a general picture of the systemic inflammatory response. Radiological imaging, typically initiated with plain X-rays, was supplemented by computed tomography (CT) and magnetic resonance imaging (MRI) when necessary. These modalities offered crucial insights into cortical destruction, intraosseous sequestra, and the integrity of surrounding soft tissues.

To determine the vascular status of the affected limb, patients underwent either computed tomography angiography (CTA) or duplex ultrasonography. This step was essential in planning for local or free flap coverage, as compromised perfusion would have significantly impacted flap viability and healing. Intraoperatively, standardized deep-tissue cultures were obtained at debridement to tailor antibiotic therapy. Infection staging was performed using the Cierny–Mader [6] system to guide the debridement and stabilization strategy.

### 2.4. Surgical Protocol

All patients in this study were managed according to a consistent and structured three-stage surgical approach, reflecting contemporary best practices in the treatment of post-traumatic osteomyelitis.

Stage 1—Source control: meticulous radical debridement of all nonviable bone and soft tissue; removal of compromised hardware; conversion to external fixation when necessary; copious irrigation. Negative-pressure wound therapy (NPWT) was used selectively as a temporizing adjunct prior to definitive coverage, consistent with mixed but supportive evidence in severe open fractures.

Stage 2—Antibiotics: perioperative broad coverage narrowed to culture-directed regimens; typical IV duration 4–6 weeks with transition to oral therapy when appropriate, in line with contemporary guidance [4].

**Figure 1:**
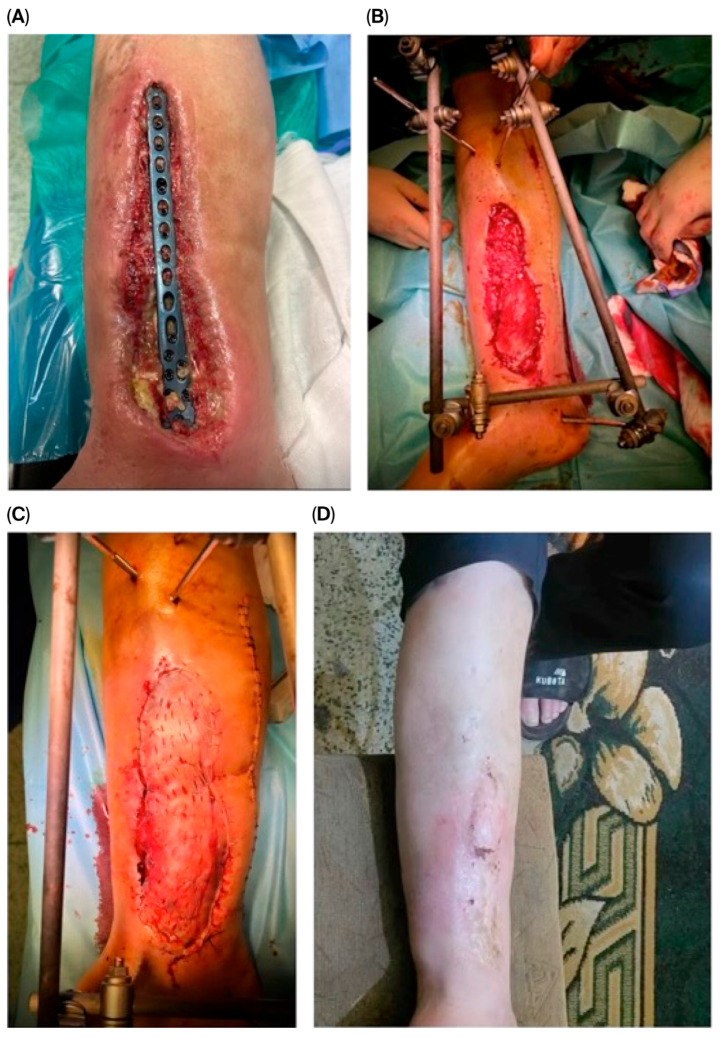
A 76-year-old male patient with an open tibial fracture initially treated surgically at an outside institution was admitted eight weeks postoperatively with a soft tissue defect over the tibial shaft, exposed hardware, signs of localized osteomyelitis, and soft tissue necrosis (**A**). The fixation material was removed, and extensive necrectomy was performed (**B**). The bone was covered using two pedicled muscle flaps, from the soleus and the medial head of the gastrocnemius (**C**), followed by coverage with a split-thickness skin graft. Due to fracture instability, the internal fixation was replaced with an external fixator. At 12 weeks postoperatively, complete wound closure (**D**) was achieved, and the patient regained independent ambulation without the need for assistive devices.

**Figure 2:**
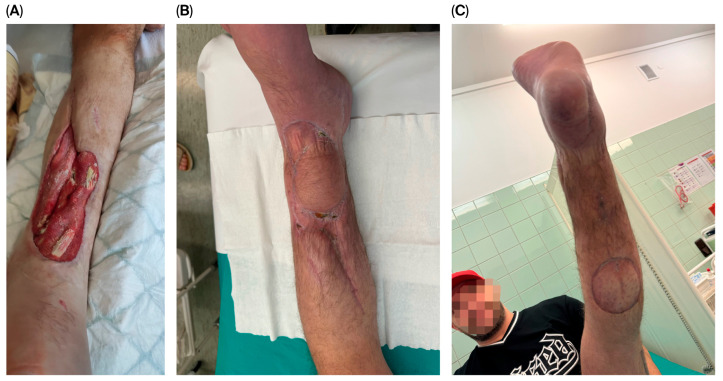
A 39-year-old male patient was referred from another hospital with a large soft tissue defect in the distal third of the lower leg (**A**) following a traffic accident. The patient also sustained injury to the posterior tibial vessels. At the primary facility, a bypass using the great saphenous vein was performed to restore continuity of the posterior tibial artery, along with a free latissimus dorsi flap. Unfortunately, both procedures failed. The patient was qualified for a below-knee amputation, which he refused. Two months after the trauma, he was admitted to our center. A simple reverse sural flap was used to achieve complete coverage of the exposed tibia, and the remaining soft tissue defect was reconstructed with a split-thickness skin graft (**B**,**C**).

**Figure 3:**
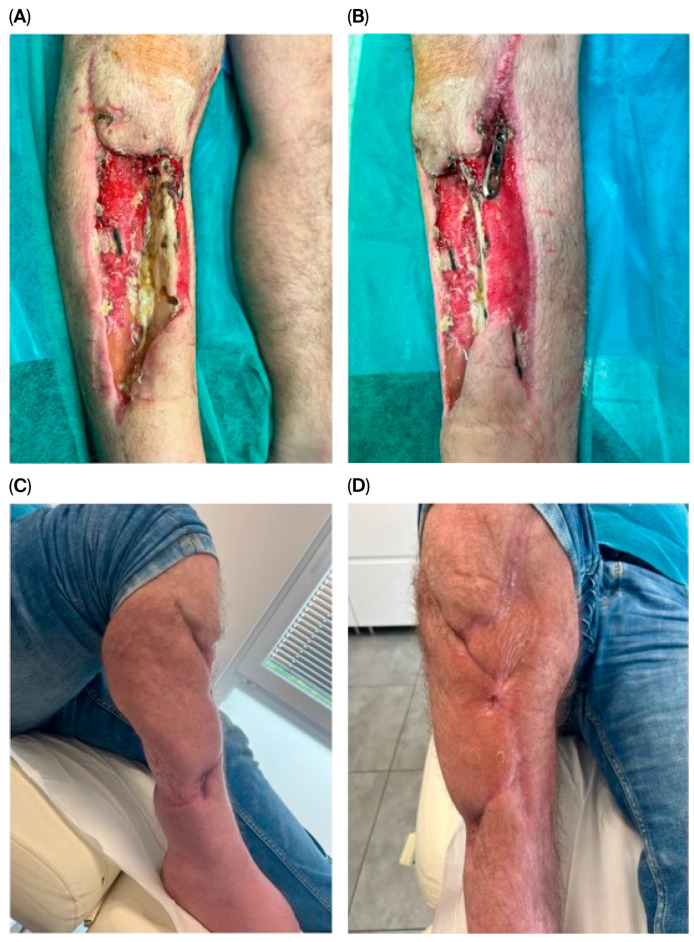
A 42-year-old male patient with a crush injury of the lower leg complicated by compartment syndrome presented with extensive exposure of the tibia and fibula, as well as underlying fixation hardware (**A**,**B**). Due to the severity of the soft tissue loss and poor local vascular quality, free flap reconstruction was not feasible. A multi-flap approach was employed, utilizing a medial gastrocnemius muscle flap, a hemisoleus muscle flap, and a proximally based sural fasciocutaneous flap. Following complete wound healing (**C**,**D**), the patient developed an Achilles tendon contracture and subsequently underwent ankle arthrodesis. At final follow-up, the patient was ambulatory without the need for orthopedic assistive devices.

**Figure 4:**
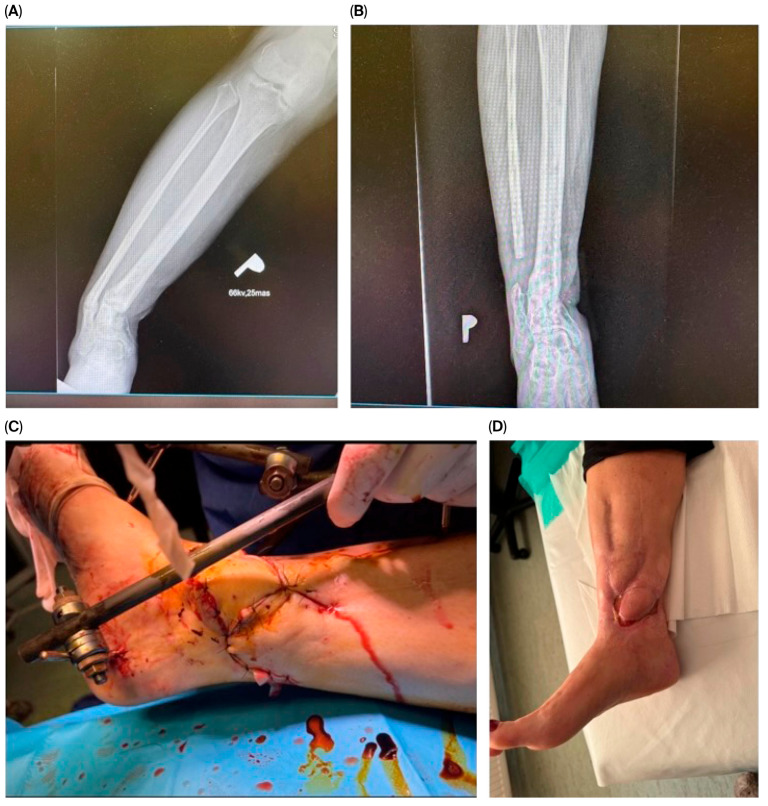
A 42-year-old female patient presented with chronic osteomyelitis, a draining bone sinus, and nonunion of the distal tibial metaphysis 1.5 years after an open fracture treated at an outside institution. Extensive debridement was performed, followed by anatomical realignment of the tibia. Partial resection of the fibula was necessary to achieve proper tibial axis correction (**A**). Soft tissue coverage was achieved using a free (MFC) medial femoral condyle flap with a skin paddle harvested from the ipsilateral limb (**C**). The vascular pedicle was anastomosed to the posterior tibial vessels. Following fracture reduction, an external fixator was applied (**D**) and maintained for 12 weeks until bone union was achieved (**B**). The patient regained full ambulatory function without the need for assistive devices.

Stage 3—Definitive reconstruction: flap selection was individualized by defect zone, contamination burden, vascular status, and host factors. Muscle flaps (Figure 1) were favored for deep dead space/high bacterial load; fasciocutaneous options (Figure 2) for superficial/cleaner defects in the distal leg. In some cases, multiple solutions were combined, including even two pedicled muscle flaps along with a pedicled fasciocutaneous flap (Figure 3). Free flap transfer was used for large, composite, or previously failed reconstructions. Vascularized bone flaps (MFC, DCIA) were used for recalcitrant nonunion or segmental bone loss in an infected bed (Figure 4).

The vascular status of recipient vessels and the patient’s comorbidities were also considered when choosing the flap, ensuring that reconstructive decisions supported both healing and long-term function.

### 2.5. Data Collection and Outcomes

To capture the full clinical trajectory of each patient, a comprehensive set of variables was collected and analyzed. This included basic demographic information such as age, sex, and medical history, with particular attention to comorbidities known to affect wound healing, i.e., diabetes, peripheral arterial disease, smoking, and immunosuppression. Each case was carefully classified by defect location, distinguishing between proximal, middle, and distal tibial involvement, given the reconstructive implications of each zone.

The details of the reconstruction were recorded in depth. For each patient, the type of flap employed, the donor site, and, where applicable, the ischemia time during microsurgical free flap transfer were documented. This allowed for subsequent analysis of flap-related outcomes and correlations with complication rates.

Complications were tracked systematically. These included both minor and major events such as partial or total flap necrosis, recurrence of infection, wound dehiscence, or the need for surgical revision. Functional outcomes were also a primary focus: postoperative ambulation status, the durability of limb salvage, and any subsequent reoperations were documented. The follow-up period ranged from six months to two years, ensuring that both early and intermediate outcomes could be meaningfully assessed.

By combining clinical, surgical, and functional data, this study aimed to provide a nuanced picture of reconstructive success and to inform decision-making in similarly complex cases of chronic lower limb osteomyelitis.

### 2.6. Statistical Analysis

Given sample size, analyses were descriptive, with 95% binomial CIs (Wilson) for key proportions. We summarized major flap complications (Table 2) and performed an exploratory Fisher’s exact test for association. All *p*-values are nominal and hypothesis generating (Table 3).

Table 2 details the reconstructive strategies undertaken, stratified by flap type, along with major flap complications. This analysis shows that reverse/pedicled sural flaps carried a higher rate of partial or total necrosis (33.3%), in contrast with hemisoleus, gastrocnemius, peroneus brevis, and free flaps (anterolateral thigh flap “ALT”, medial femoral condyle flap “MFC”, deep circumflex iliac artery bone flap “DCIA”, radial forearm free flap “RFFF”) where no major necrosis was observed. This is consistent with published concerns about venous congestion and tip necrosis in sural flaps. These findings reinforce the importance of careful patient selection and perioperative monitoring when using sural flaps.

Table 3 summarizes patient-level outcomes including flap survival, infection resolution, amputation, ambulation, and return to work. Flap survival and infection resolution were both 90% (95% “Confidence Interval” CI ≈ 0.70–0.97), while the amputation rate was 5%. All limb-salvaged patients were independently ambulatory at follow-up, and 73% of previously employed patients returned to work. These outcomes are aligned with international benchmarks and highlight the functional impact of a structured orthoplastic approach.

## 3. Results

### 3.1. Cohort Characteristics

A total of 20 adults (15 men, 5 women; mean age 53.6 years [range 36–82]) presented with chronic PTO (>6 weeks from index trauma). Mechanisms: road-traffic injury (n = 12), fall from height (n = 5), industrial crush (n = 3). Comorbidities: diabetes (n = 6), peripheral vascular disease (n = 4), current smokers (n = 11), obesity (BMI ≥ 30; n = 7). Prior internal fixation had been performed in 14 patients.

All patients presented with well-established, chronic infections, defined as persisting for more than six weeks following the initial trauma. For all of these cases, prior attempts at surgical management had been undertaken at other institutions but had proved unsuccessful, either due to incomplete debridement, wound breakdown, or persistent infection.

Several comorbidities commonly associated with impaired wound healing and increased infection risk were noted in this cohort. Diabetes mellitus was present in six patients, while four suffered from peripheral vascular disease affecting lower limb perfusion. Chronic smoking was reported by eleven individuals, and seven were classified as obese with a body mass index exceeding 30. Fourteen patients had undergone prior internal fixation, with hardware either retained or subsequently removed due to exposure or infection.

The anatomical distribution of the defects revealed a clear predominance of involvement in the middle and distal thirds of the tibia, regions particularly prone to soft tissue breakdown due to limited vascularization and minimal soft-tissue envelope. In all cases, the bone was visibly exposed at the time of assessment. Notably, seven patients exhibited radiographic or intraoperative evidence of bony sequestra, indicating long-standing osteonecrosis.

This clinical portrait highlights not only the complexity of the defects encountered but also the systemic fragility of the patient population, a factor that influences both surgical planning and long-term outcomes. The majority of defects involved the middle or distal third of the tibia, with exposed bone in all cases.

Microbiology: Intraoperative cultures were positive in all cases: *S. aureus* (n = 14, including 5 MRSA), *P. aeruginosa* (n = 5), *E. faecalis* (n = 3), and mixed aerobic/anaerobic flora (n = 4).

### 3.2. Flap Selection and Distribution

**Figure 5:**
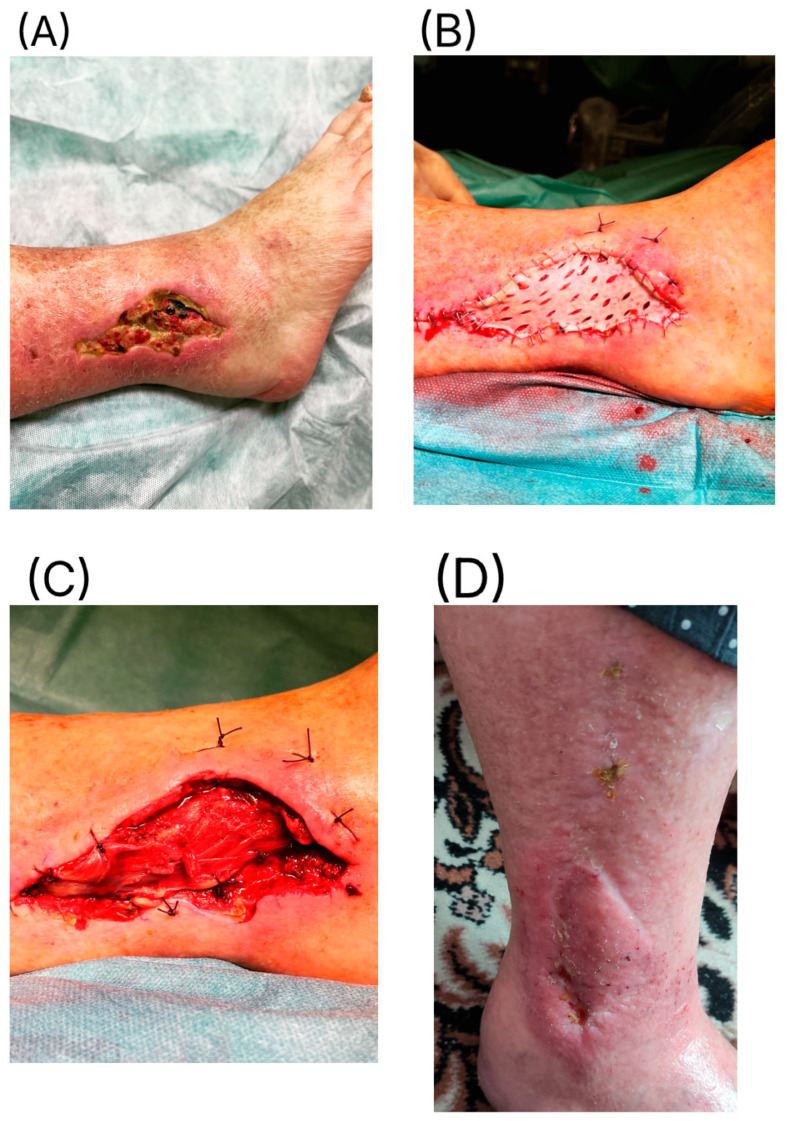
An 82-year-old female patient presented seven weeks after open reduction and internal fixation of a lateral malleolar fracture, initially treated at an outside institution. She was admitted with a soft tissue defect over the lateral ankle, exposed hardware, signs of localized osteomyelitis, and soft tissue necrosis (**A**). The fixation material was removed, followed by extensive necrectomy. The bone was covered with a proximally based peroneus brevis muscle flap (**B**), over which a split-thickness skin graft was applied (**C**). At 10 weeks postoperatively, complete wound closure was achieved (**D**). The patient regained ambulatory function without the need for assistive devices.

The choice of flap for each reconstruction was guided by a combination of anatomical considerations, defect complexity, vascular status, and previous surgical history. Local and regional flaps formed the backbone of most reconstructions; however, free tissue transfer was used in more complex cases requiring larger volumes of tissue or composite reconstruction. Among the patients, eight received distally based sural flaps, one received a pedicled sural flap, seven received hemisoleus muscle flaps, two received medial gastrocnemius muscle flaps, two received peroneus brevis muscle flaps (Figure 5), and six received different free flaps. Reconstruction was performed using more than one flap or a single flap in 7 cases (Figure 3) and 13 cases (Table 1), respectively.

Among the six free flap procedures performed, the anterolateral thigh free flap was the most frequently used (three cases) because of its versatility and large skin paddle. One patient underwent reconstruction of bone nonunion at the distal 1/3 of the tibia using a medial femoral condyle osteoperiosteal flap with a skin island (Figure 4), which was chosen for its reliable vascular anatomy and excellent contour matching in smaller bone defects. One patient received a deep circumflex iliac artery osteocutaneous flap, which provided both robust bone and soft-tissue coverage for a particularly extensive anteromedial tibial defect, and one patient with metatarsal osteomyelitis and dorsal soft-tissue loss underwent reconstruction using a free radial forearm flap.

The average ischemia time for the free flaps was 78 min, with a range from 65 to 110 min. Recipient vessels were carefully selected based on preoperative imaging and intraoperative evaluation. In four cases, the anterior tibial artery served as the recipient artery, while the posterior tibial artery was used in the remaining two. All venous anastomoses were completed using end-to-end techniques with microsurgical coupler systems to minimize technical error and reduce operative time. All free flap procedures were performed by a board-certified senior microsurgeon with more than 20 years of dedicated experience in limb reconstruction.

### 3.3. Complications, Infection Control, and Limb Salvage

Overall flap survival was 90%. Major flap complications occurred in three reconstructions—all within the sural cohort (partial necrosis in two; complete loss in one that required reoperation). Two additional patients had minor wound dehiscence; no perioperative thromboembolic or cardiopulmonary events occurred.

Infection resolved in 18/20 patients (90%; 95% CI ≈ 0.70–0.97). One patient with persistent nonunion underwent below-knee amputation at his request (limb-level amputation rate 5%; 95% CI ≈ 0.9–23.6%). At final review (mean 10 months, range 6–24), all limb-salvaged patients were independently ambulant; 11/15 previously employed patients returned to work within 6–12 months.

## 4. Discussion

Reconstructive management of post-traumatic osteomyelitis requires more than technical proficiency in surgery. It demands a comprehensive understanding of the interplay between local pathology and systemic health, a nuanced approach to infection control, and a long-term vision for restoring limb function and quality of life. The process is as much about decision-making and timing as it is about muscle flaps and microsurgical technique. At its core, post-traumatic osteomyelitis is an infected microenvironment. The infected bone is often riddled with avascular, necrotic segments, a fertile ground for bacterial colonization. The pathogens, most often Staphylococcus aureus and Pseudomonas aeruginosa, thrive in the biofilms that form on devitalized bone and hardware, effectively shielding them from both the host immune response and systemic antibiotics. Chronic cases may develop complex architecture, including sinus tracts, involucrum, and sequestra, hallmarks of a deep-seated, self-sustaining infection [1,2]. These features make it abundantly clear that antibiotic therapy alone is insufficient. Only complete surgical eradication of infected and nonviable tissue offers a chance for definitive cure. Vascularized soft tissue does more than fill a gap, it transforms the local biological environment. Well-perfused muscle or fasciocutaneous tissue allows oxygen, immune cells, and antibiotics to access the site of infection, all of which are critical for bacterial clearance and wound healing. Numerous studies have demonstrated that oxygen tension and leukocyte activity are significantly increased in wounds covered with vascularized muscle, enhancing the likelihood of infection resolution [4,5,6].

Our experience reinforces the continued value of local muscle flaps, such as the medial gastrocnemius, hemisoleus, and peroneus brevis, for managing moderate-sized defects, particularly in the proximal and middle thirds of the lower limb. Among them, the hemisoleus flap stands out for its consistent anatomy, minimal donor morbidity, and ability to contour well over diaphyseal bone. It remains a reliable workhorse in our reconstructive arsenal [7]. Defects of the distal lower limb present a particular challenge due to the thin soft-tissue envelope and limited options for local muscle coverage. In this anatomical zone, the distally based sural flap continues to offer a dependable solution, especially when free flap capabilities are not readily available or the patient is not a candidate for prolonged surgery. Although partial flap necrosis can occur, typically due to venous congestion, this risk can be mitigated through careful attention to perforator anatomy and tension-free inset [8,9].

Our data confirm the utility of this procedure in selected patients with smaller to moderate-sized wounds in the distal third of the leg. In more complex cases, where defects are extensive, previously irradiated, involve both soft-tissue and bone loss, or follow failed reconstructions, free flap transfer becomes not only appropriate but essential. Among our patients, six required free flaps, reflecting the need for high-volume composite reconstruction. The anterolateral thigh (ALT) flap remains our preferred option for soft-tissue coverage due to its generous skin paddle, long vascular pedicle, and low donor-site morbidity. For cases involving segmental bone loss or nonunion within an infected bed, osteocutaneous free flaps like the medial femoral condyle (MFC) and deep circumflex iliac artery (DCIA) flaps offer an effective solution by providing both structural support and biological viability [10,11,12]. However, these microsurgical procedures require not only technical expertise but also careful patient selection and intensive postoperative monitoring. Institutional experience plays a critical role in minimizing flap loss and optimizing long-term outcomes. Not every limb can, or should, be salvaged. Our 5% amputation rate is comparable to those reported by major limb reconstruction centers, reflecting the balance between ambition and realism. The decision to proceed with amputation is never taken lightly, but it should not be viewed as a failure. Rather, it is sometimes the most rational, patient-centered option, particularly when faced with recurrent infection, nonunion, intractable pain, or psychological exhaustion. Objective tools such as the Limb Salvage Index (LSI) and the Mangled Extremity Severity Score (MESS) can assist in structuring this decision, but they must be integrated with clinical judgment and patient preference [13].

Studies by Jupiter and Dodd have shown that functional outcomes with modern prosthetics can match, or even exceed, those achieved with chronically salvaged limbs plagued by pain and immobility [14,15]. In these situations, success should be measured not in terms of anatomical preservation, but by restored function and quality of life. Timing is perhaps the most underappreciated variable in limb salvage. In our institutional algorithm, we emphasize intervention within 72 h of clinical deterioration or wound breakdown. This “golden window” for reconstruction is associated with significantly lower infection recurrence and better functional outcomes in several published series [16,17].

Equally crucial is the collaborative nature of modern PTO management. Our best results came from early, coordinated input by orthopedic surgeons, plastic surgeons, and infectious disease specialists. When all these parties are engaged early and work synergistically, outcomes are not simply optimized but often transformational [18].

The outcomes of our series correspond closely with those reported in the broader literature. Overall flap survival approached 90 percent, while the amputation rate remained at five percent, both figures aligning with benchmarks described in large series of complex lower-extremity reconstructions. Within our cohort, reverse and pedicled sural flaps demonstrated a higher rate of major necrosis, a finding consistent with reports that highlight venous congestion and distal tip ischemia as inherent risks of this technique. By contrast, muscle flaps and free transfers performed favorably, reinforcing the well-established reliability of these options, though we recognize that the modest size of our series necessitates caution in drawing definitive comparisons [18,19,20,21].

Several features strengthen the validity of this study. The prospectively maintained database ensured the accuracy and completeness of clinical records, while the use of a standardized staged protocol minimized variability in the timing and sequence of interventions. Our armamentarium was broad and included vascularized bone flaps, thereby allowing a tailored response to heterogeneous defects. Furthermore, all procedures were performed by a single microsurgical team, lending consistency to operative technique and perioperative management. At the same time, limitations must be acknowledged.

The retrospective design inherently reduces the power of causal inference, and the relatively small cohort size limited our ability to conduct multivariable analyses. The absence of a control group further restricts direct comparison with alternative strategies. Patient heterogeneity, including the diversity of defects and prior treatments, introduces additional variability. The follow-up period, which ranged from six to twenty-four months, though sufficient to capture early outcomes, remains too short to fully assess long-term durability. Finally, the lack of systematically collected and validated patient-reported outcomes, such as the Lower Extremity Functional Scale, is a significant shortcoming that we intend to address in future prospective work.

Despite these limitations, the proposed algorithm demonstrates adaptability across different healthcare environments. In tertiary centers with access to microsurgical expertise, free flaps greatly expand the range of reconstructive options and should be considered a cornerstone in complex cases. In more resource-constrained settings, however, regional composite strategies—such as the combination of muscle and sural flaps—remain effective and reproducible. A common framework that unites these diverse approaches is the use of the Cierny–Mader [6] staging system, which provides a standardized language for classification and allows treatment decisions to be compared and harmonized across institutions worldwide.

Looking forward, the field requires rigorous prospective validation of algorithmic pathways in multicenter settings to ensure reproducibility beyond individual institutional experience. A particular priority will be the standardized collection of functional outcomes through instruments such as the LEFS or PROMIS PF, which can provide objective insight into the true quality of recovery from the patient’s perspective. Comparative effectiveness studies that directly examine the relative merits of muscle, fasciocutaneous, and free-tissue options in the context of post-traumatic osteomyelitis are also needed, as the current evidence remains largely descriptive. Equally important is the integration of emerging biologic strategies—including antibiotic carriers, bioactive bone substitutes, and other orthobiologics—into comprehensive orthoplastic care pathways. Such integration offers the possibility of advancing beyond structural salvage toward biologically optimized, durable, and patient-centered reconstruction [22,23,24,25,26,27,28].

## 5. Conclusions

Post-traumatic osteomyelitis of the lower limb remains one of the most formidable and multidimensional challenges in contemporary reconstructive surgery. It is a pathology that tests not only surgical skill but also clinical judgment, interdisciplinary coordination, and the surgeon’s understanding of long-term patient needs. Treating it successfully is not simply a matter of clearing infection, it is about restoring anatomy, enabling function, and ultimately helping the patient reclaim autonomy and quality of life.

In a consecutive cohort of PTO patients managed with a structured, comorbidity- and perfusion-informed algorithm, we observed 90% infection resolution, 90% flap survival, 5% amputation, and universal independent ambulation among limb-salvaged patients at ~10 months. These outcomes support early orthoplastic coordination, radical debridement, and individualized flap selection, while underscoring the need for larger prospective studies and routine validated functional metrics.

## Figures and Tables

**Table 1:** Cohort characteristics.

	Sex	Age	Mechanism of Injury	Defect Location	Type of Flap Reconstruction	Complications
1	M	39	motorcycle accident	distal 1/3 of the leg	hemisoleus muscle flap, reverse—flow sural artery flap	none
2	M	42	crush injury	proximal 2/3 of the leg	medial gastrocnemius muscle flap, hemisoleus muscle flap, pedicled sural flap	none
3	M	49	crush injury	distal 1/3 of the leg	reverse—flow sural artery flap	none
4	F	42	car accident	distal 1/3 of the leg	MFC	none
5	M	76	fall from high	distal 2/3 of the leg	medial gastrocnemius muscle flap, hemisoleus muscle flap	none
6	M	72	fall from high	proximal 1/3 of the leg	hemisoleus muscle flap, reverse—flow sural artery flap	none
7	F	82	bicycle accident	distal 1/3 of the leg	peroneus brevis muscle flap	none
8	M	46	car accident	middle third of the leg	ALT	none
9	M	67	car accident	middle third of the leg	hemisoleus muscle flap, reverse—flow sural artery flap	failure of sural flap
10	M	36	fall from high	middle third of the leg	reverse—flow sural artery flap	none
11	M	72	fall from high	distal 1/3 of the leg	peroneus brevis muscle flap	none
12	F	44	bicycle accident	proximal 1/3 of the leg	ALT	none
13	M	46	crush injury	foot	RFFF	none
14	M	64	bicycle accident	foot	DCIA	pathological fracture
15	F	39	motorcycle accident	distal 2/3 of the leg	reverse—flow sural artery flap	partial flap necrosis
16	M	41	car accident	proximal 1/3 of the leg	ALT	none
17	M	39	motorcycle accident	distal 1/3 of the leg	reverse—flow sural artery flap	none
18	F	42	car accident	distal 1/3 of the leg	reverse—flow sural artery flap	partial flap necrosis
19	M	71	bicycle accident	middle third of the leg	hemisoleus muscle flap, reverse—flow sural artery flap	none
20	M	63	fall from high	distal 1/3 of the leg	hemisoleus muscle flap, reverse—flow sural artery flap	none

**Table 2:** Reconstruction by flap category and complications.

Flap Category	Reconstructions, n	Major Flap Necrosis, n (%)
Reverse/pedicled sural	9	1 total (11) and 2 (22) minor necrosis
Hemisoleus	7	0 (0)
Medial gastrocnemius	2	0 (0)
Peroneus brevis	2	0 (0)
Free flaps (ALT, MFC, DCIA, RFFF)	6	0 (0)
Total	26	1 (3,8)

**Table 3:** Key outcomes.

Outcome	Value (95% CI Where Applicable)
Flap survival (patient-level)	96.2%
Infection resolution	18/20 (90%; 95% CI ≈ 0.70–0.97)
Amputation	1/20 (5%; 95% CI ≈ 0.9–23.6%)
Ambulation at last follow-up	19/19 limb-salvaged patients independent
Return to work (among employed)	11/15 within 6–12 months

## Data Availability

Data is contained within the article.

## References

[1] Lew D.P., Waldvogel F.A. (2004). Osteomyelitis. Lancet.

[2] Bury D.C., Rogers T.S., Dickman M.M. (2021). Osteomyelitis: Diagnosis and Treatment. Am. Fam. Physician.

[3] Gausden E.B., Ziran N.M., Pape H.C. (2021). Diagnosis and Management of Chronic Osteomyelitis. J. Am. Acad. Orthop. Surg..

[4] Morgenstern M., Kuehl R., Zalavras C.G., McNally M., Zimmerli W., Burch M.A., Vandendriessche T., Obremskey W.T., Verhofstad M.H.J., Metsemakers W.J. (2021). The influence of duration of infection on outcome of debridement and implant retention in fracture-related infection. Bone Joint J..

[5] Godina M. (1986). Early microsurgical reconstruction of complex trauma of the extremities. Plast. Reconstr. Surg..

[6] Cierny G., Mader J.T., Penninck J.J. (2003). A clinical staging system for adult osteomyelitis. Clin. Orthop. Relat. Res..

[7] Chan J.K.K., Ferguson J.Y., Scarborough M., McNally M.A., Ramsden A.J. (2019). Management of Post-Traumatic Osteomyelitis in the Lower Limb: Current State of the Art. Indian J. Plast Surg..

[8] McNally M.A., Ferguson J.Y., Lau A.C., Diefenbeck M., Scarborough M., Ramsden A.J., Atkins B.L. (2016). Single-stage treatment with gentamicin-loaded ceramic carrier. J. Bone Jt. Surg. Am..

[9] Khouri R.K., Shaw W.W. (1989). Lower-extremity free-flap reconstruction: 304 consecutive cases. J. Trauma.

[10] Baumeister S.P., Spierer R., Erdmann D., Levin L.S., Germann G. (2003). Sural flaps for lower leg/foot: 124 cases. Plast. Reconstr. Surg..

[11] Kneser U., Bach A.D., Polykandriotis E., Kopp J., Horch R.E. (2005). Delayed reverse sural flap. Plast. Reconstr. Surg..

[12] Prabhakar S., Shah H., Hameed S. (2017). Chronic tibial osteomyelitis review. Clin. Podiatr. Med. Surg..

[13] Loja M.N., Sammann A., DuBose J., Li C.S., Liu Y., Savage S., Scalea T., Holcomb J.B., Rasmussen T.E., Knudson M.M. (2017). The mangled extremity score and amputation: Time for a revision. J. Trauma Acute Care Surg..

[14] Marrara G., Zampogna B., Schick V.D., Larizza L., Rizzo P., Sanzarello I., Nanni M., Leonetti D. (2025). Post-Traumatic Segmental Tibial Defects Management: A Systematic Review of the Literature. Appl. Sci..

[15] Johansen K., Daines M., Howey T., Helfet D., Hansen S.T. (1990). Predicting amputation after lower-extremity trauma (MESS). J. Trauma.

[16] Tyner C.E., Kisala P.A., Slotkin J., Cohen M.L., Cancio J.M., Pruziner A.L., Dearth C.L., Tulsky D.S. (2025). Health-related quality of life after major extremity trauma: Qualitative research with military service members and clinicians to inform measurement of patient-reported outcomes. Qual. Life Res..

[17] Jha R.K., Jayaram P.V., Shankaran R., Pillai H.J. (2023). Salvage of a severely mangled limb following traumatic injury. BMJ Case Rep..

[18] Sharma H., Babhulkar S., Michael A. (2021). Early soft-tissue coverage prevents chronic osteomyelitis. Int. Orthop..

[19] Mehta D.D., Leucht P. (2024). Prevention and treatment of osteomyelitis after open tibia fractures. OTA Int..

[20] Gopal S., Majumder S., Batchelor A.G., Knight S.L., De Boer P., Smith R.M. (2000). Fix and flap: The radical orthopaedic and plastic treatment of severe open fractures of the tibia. J. Bone Jt. Surg. Br..

[21] Binkley J.M., Stratford P.W., Lott S.A., Riddle D.L. (1999). The Lower Extremity Functional Scale (LEFS): Scale development, measurement properties, and clinical application. North American Orthopaedic Rehabilitation Research Network. Phys. Ther..

[22] Stannard J.P., Volgas D.A., Stewart R., McGwin G., Alonso J.E. (2009). Negative pressure wound therapy after severe open fractures: A prospective randomized study. J. Orthop. Trauma.

[23] Costa M.L., Achten J., Bruce J., Tutton E., Petrou S., Lamb S.E., Parsons N.R., UKWOLLF Collaboration (2018). Effect of Negative Pressure Wound Therapy vs. Standard Wound Management on 12-Month Disability Among Adults With Severe Open Fracture of the Lower Limb: The WOLLF Randomized Clinical Trial. JAMA.

[24] Tripathee S., Basnet S.J., Lamichhane A., Hariani L. (2022). How Safe Is Reverse Sural Flap?: A Systematic Review. Eplasty.

[25] Özdemir A., Odabaşı E., Acar M.A. (2022). The free medial femoral condyle periosteal flaps for the treatment of recalcitrant upper limb long bones nonunion. Ulus. Travma Acil Cerrahi Derg..

[26] Matsumoto T., Tsumura T., Kishimoto K., Sano H., Doi K., Matsushita M., Murakami H. (2020). Sequential chimeric free deep circumflex iliac artery bone flap and superficial circumflex iliac artery perforator flap from the same site for one-stage reconstructions of severe hand injury: A report of two cases. JPRAS Open.

[27] Maimaiti X., Liu K., Yusufu A., Xie Z. (2024). Treatment of tibial bone defects caused by infection: A retrospective comparative study of bone transport using a combined technique of unilateral external fixation over an intramedullary nail versus circular external fixation over an intramedullary nail. BMC Musculoskelet. Disord..

[28] Haykal S., Roy M., Patel A. (2018). Meta-analysis of Timing for Microsurgical Free-Flap Reconstruction for Lower Limb Injury: Evaluation of the Godina Principles. J. Reconstr. Microsurg..

